# Detection of electrical signals in fungal mycelia in response to external stimuli

**DOI:** 10.1016/j.isci.2025.113484

**Published:** 2025-09-02

**Authors:** Matteo Buffi, Silvia Giangaspero, Valerio Foiada, Loïc Puthod, Guillaume Cailleau, Aaron J. Robinson, Julia M. Kelliher, Patrick S.G. Chain, Daniel Oberson, Markus Künzler, Saskia Bindschedler, Lorenzo Pirrami, Pilar Junier

**Affiliations:** 1 Laboratory of Microbiology, University of Neuchâtel, 2000 Neuchâtel, Switzerland; 2 iSIS Institute, HEIA-FR, HES-SO University of Applied Sciences and Arts Western Switzerland, 1700 Fribourg, Switzerland; 3 Prototypos Sarl, 1700 Fribourg, Switzerland; 4 Bioscience Division, Los Alamos National Laboratory, Los Alamos, NM 87545, USA; 5 Institute of Microbiology, Department of Biology, ETH Zürich, 8093 Zurich, Switzerland

**Keywords:** Mycology, Biophysics

## Abstract

Electrical signaling is a crucial mechanism for intercellular communication across diverse biological systems. Despite evidence of electrical activity in fungal mycelia, a standardized, reproducible method for detecting these signals is lacking. In this study, we developed a novel approach using printed circuit boards with embedded differential electrodes to record extracellular voltage fluctuations in mycelium. By incorporating a Faraday cage and short-time Fourier transform analysis, we minimized noise and extracted relevant frequency patterns. Our findings revealed electrical activity correlated with fungal growth that varied with biocide treatments. The results support the biological origin of these signals, suggesting a role in environmental adaptation. This study provides a robust framework for further exploration of fungal electrophysiology, with implications for understanding signaling mechanisms in mycelial networks.

## Highlights

• Development of a tool to study extracellular voltage fluctuations in fungi
• Features minimizing experimental artifacts reducing abiotic and environmental noise
• Application of advanced signal analysis for extraction of frequency patterns
• Robust foundation for further exploration of electrical signaling in fungi

## Graphical abstract

**Figure:**
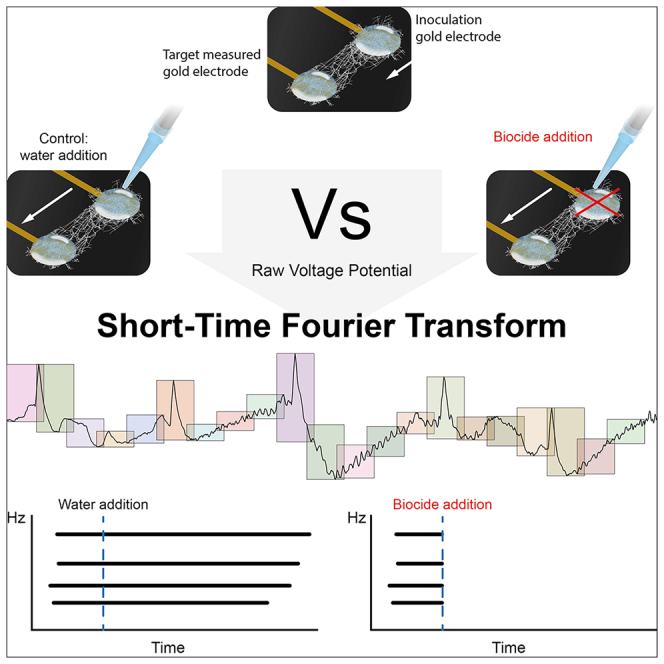

## Introduction

Electrical signaling as a mechanism of inter- and intracellular communication is used by a wide diversity of organisms including animals, plants, and microorganisms.^1^^,^^2^^,^^3^ Since Luigi Galvani’s discovery of so-called “animal electricity,”^4^ propagating action potentials have been identified as the principal communication mechanism in the central nervous system of animals.^5^ Charles Darwin also observed action-potential-like signals in one of his favorite plants, *Dionaea muscipula*,^6^ and it is now widely accepted that many physiological functions in plants rely on action potentials with the same characteristics of those of animals.^7^^,^^8^^,^^9^^,^^10^ However, given the fundamental differences between animals and plants, additional types of electrical signaling processes have been recognized only in the case of the latter. In plants, variation and systemic potentials are long-distance intercellular electrical signals generated by transient membrane depolarization that coordinate functional responses under stress conditions.^8^^,^^9^^,^^10^^,^^11^ Electrical signaling has also been recognized as a means of communication in bacterial biofilms, where it facilitates nutrient sharing and aids in the recruitment of distant cells.^3^ The diversity of examples of electrical signaling across the entire tree of life suggests the universality of this mechanism of communication. Electrical signaling is the result of a fundamental function of cell membranes: the selective transport of charged molecules that creates a disequilibrium of charge across the membrane, i.e., a membrane potential.^12^ This electrochemical gradient is essential for energy production^13^ but it also enables communication between adjacent cells, as it can be propagated along the cell membrane via voltage-gated ion channels.^14^

The mycelium of filamentous fungi is a highly plastic network of interconnected cells or hyphae that allows fungi to adapt dynamically to uneven or ephemeral resources in complex environments such as soil or other habitats.^15^^,^^16^ The architecture of this network depends on multiple factors, but ultimately on the polar growth of hyphal tips, hyphal branching, and hyphal fusion (anastomosis).^17^ While the vegetative hyphae of basal fungal clades (i.e., Mucoromycota) are not compartmentalized, hyphae of higher fungi (Dikarya) contain regular septa with pores that regulate exchange and transport among compartments and play a role in differentiation and reproduction.^18^^,^^19^ Septation also prevents loss of cytoplasm after hyphal damage^20^^,^^21^ or in response to attack^22^^,^^23^ but restricts the propagation of chemical signals via cytoplasmic bulk flow. In contrast, the continuity of the cellular membrane, even across plugged septal pores, offers a mechanism by which electrical signaling could allow for communication between distant hyphal compartments. Moreover, the membrane is surrounded by the cell wall that is comprised of components that can act as electrical insulators analogously to myelin in neurons.^24^^,^^25^^,^^26^

Filamentous fungi are known to produce electrical currents in their hyphal tips.^27^^,^^28^ Pioneering work using internal electrodes has shown action-potential-like spikes in rhizomorphs or loose mycelium of *Armillaria bulbosa* and *Pleurotus ostreatus* exposed to a wood block (complex source of carbon) or in response to mechanical pressure.^29^ These currents or membrane potential changes were low in voltage (nV to μV).^28^^,^^29^^,^^30^ More recently, several studies using needle electrodes inserted into fruiting bodies^31^^,^^32^^,^^33^^,^^34^^,^^35^^,^^36^ recorded action-potential-like spikes in various fungi and suggested a type of species-specific frequency code in the signal.^32^ Even though these studies offer an initial insight into the production and potential role of electrical signaling in filamentous fungi, a reproducible method to detect these signals and confirm their biological origin is still lacking.^37^ Furthermore, since not all fungi produce macrostructures such as fruiting bodies or rhizomorphs, it is necessary to confirm the existence of membrane potential fluctuations at the level of vegetative mycelium or single hyphae and to extend the observations beyond Basidiomycetes, which have been the subject of most studies conducted thus far.

## Results

### Development of an experimental setup for recording of electrical signals in filamentous fungi

**Figure 1:**
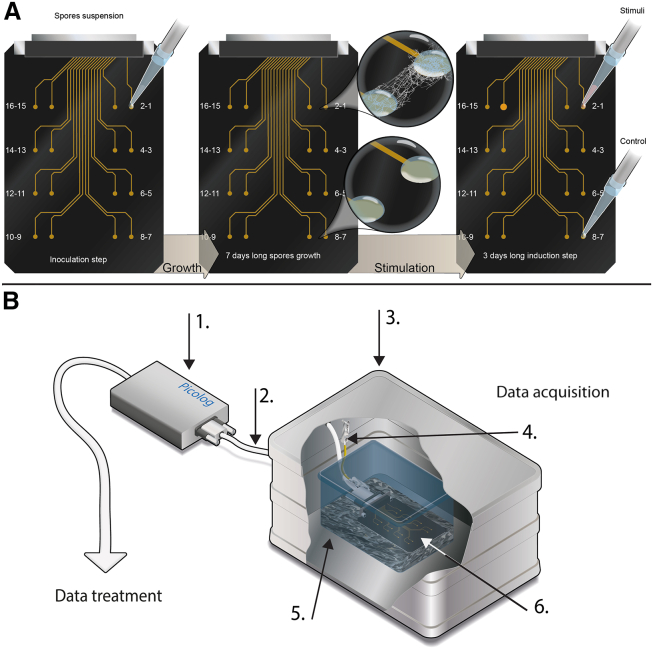
Schemes showing the development of the fungal potential card (FPC), an optimized system for the recording of electrical signals in filamentous fungi (A) The system is based on printed circuit boards (PCBs) with pairs of embedded differential electrodes to measure voltage changes. In this system, the fungus is inoculated as a spore suspension (left image) on one of the electrodes. The fungus is allowed to grow and colonize the second electrode as shown in the bubble in the middle of the figure. After this, the effect of different stimuli can be assessed (right image). (B) For recording, the system is placed in a custom-made incubator. The components of the incubation system included (1) data logger; (2) shielded cable; (3) Faraday cage; (4) crocodile head cable connected to the PCB; (5) filter for gas exchange; and (6) moistened vermiculite at the bottom to maintain optimal growth conditions.

We designed the fungal potential card (FPC) for the extracellular recording of electrical signals produced by filamentous fungi. The FPCs are printed circuit boards with pairs of embedded differential electrodes to measure extracellular changes in voltage of a growing mycelium (Figures 1 and S1). Droplets containing medium with or without fungal spores were deposited on the electrodes in a manner consistent with previously described methods.^38^ The spores were inoculated onto one of the electrodes in each pair, and, upon germination and hyphal growth, the mycelium was expected to create a connection with the second electrode in the pair. Moreover, as the system is open, stimuli can be added to validate the biological origin of the signal (Figure 1A). The FPC was placed inside an incubation chamber designed to maintain humidity and prevent evaporation of the inoculation droplets during the experiments (Figure 1B). The system was placed inside a Faraday cage to decrease external noise and artifacts. This experimental setup successfully maintained optimal conditions for growth, while also permitting the collection of nearly noise-free recordings.

### Validation of the experimental setup

To validate the method, we selected *Fusarium oxysporum*, an ascomycete that is ubiquitous in soils,^39^^,^^40^^,^^41^ has a fast growth rate, and can easily be propagated through spores, making it ideal for the inoculation approach employed in this study. As a first step, we tested the biocompatibility of the electrodes as well as the impact of the electrode size and distance between electrode pairs on hyphal growth. For this, *F. oxysporum* was grown on FPCs with different electrode sizes (1, 2, 3, and 4 mm) placed 1 cm apart (center to center). We selected gold-covered electrodes because gold is considered an inert non-toxic material.^42^^,^^43^ The smallest electrode size (1 mm) resulted in inconsistent colonization and was difficult to inoculate accurately. For larger electrode sizes (3 and 4 mm), the fungus did not consistently colonize the second electrode. In contrast, with 2 mm electrodes, the fungus was able to consistently colonize and connect the two electrodes on the FPCs, and therefore, this electrode size was selected for all of the following experiments (Figure S2).

### Development of a method for data interpretation

**Figure 2:**
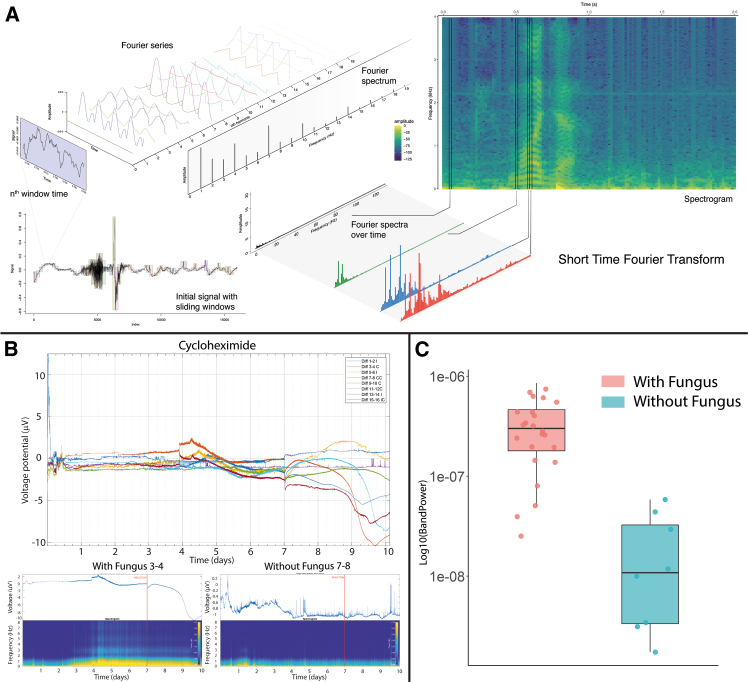
Transformation of raw signals into frequencies for data analysis (A) The signal is recorded as voltage change between the inoculated electrodes and a reference electrode. The signal is further transformed into frequencies using a short-time Fourier transform (STFT). This procedure results in a spectrogram that represents the signals’ component frequencies over time and allows for comparison against controls. (B) In the example shown here, the top panel represents the raw voltage measurements. After STFT, the pattern in the spectrogram on the left (corresponding to the inoculated electrode) is clearly distinct from the background in the uninoculated controls (right image). The change starts at 3 days after inoculation, but it is more pronounced after 4 days. (C) To quantitatively assess the difference in the STFT pattern between conditions, a power analysis was performed by integrating the power spectral density (PSD) comparing inoculated (with fungus) and non-inoculated (without fungus) electrode pairs. This difference was significant (*p* value < 0.001).

Interpretation of the recorded data can be challenging, and this was considered when analyzing the data collected on the selected 2-mm electrode FPCs. The use of a Faraday cage makes it impossible to assess the density of hyphae connected to each electrode, which is a feature required for characterization of raw voltage measurements as action-potential-like signals. Therefore, the changes in voltage were broken down to their frequency components using a short-time Fourier transform (STFT). This type of analysis results in a spectrogram that displays frequencies over time (Figure 2A). In contrast to raw recordings, which only show how the signal behaves in the time domain, STFT enables the analysis of both time and frequency simultaneously. This is particularly useful for identifying patterns in the data that may evolve or shift over time and would be hard to detect using the raw voltage data alone. For instance, the colonization of the second electrode by the growing hyphae was expected to result in a change in the STFT data pattern. To test this, we grew the fungus for seven days and recorded the voltage changes between the two electrodes. Controls without inoculation were also included in these experiments. Based on previous experiments with this isolate of *F. oxysporum*, we estimated that the mycelium would take 3–4 days to connect the pair of electrodes.^38^ The use of a Faraday cage prevented the direct monitoring of the colonization, but a change in the STFT pattern was consistently observed four days post-inoculation, which is consistent with the previously observed colonization time.^38^ The signal profiles from inoculated electrode pairs were visually distinct; in the non-inoculated controls, a background signal with a frequency below 1 Hz was recorded (yellow color in the STFT frequency). The signals from electrode pairs connected by the fungal mycelium also contained this low-frequency component prior to day four, but after this period, additional frequencies above 1.5 Hz were detected (Figure 2B). To further characterize the data, we calculated the power spectral density (PSD), which quantifies the strength or intensity of a signal at different frequencies but results in a single cumulative value. This allowed us to compare how the bulk frequency signal changed comparing the inoculated electrodes versus the control electrodes without the fungus. The results showed a 1,604% PSD increase in the inoculated electrodes compared to the non-inoculated electrodes, which was significant (Figure 2C; Table 1). This suggests that our setup records electrical signals generated by the colonizing mycelium. However, further evidence was needed to confirm the biological origin of the observed signals.

**Table 1:** Summary of the results of the power spectral density (PSD) analysis across the tested experimental conditions

Fungi vs. no fungi before induction	Replicates	Fungi mean	No fungi mean	*p* value	% change
Fungi/No fungi	24 f/8 nf	3.44429E-07	2.0204E-08	0.000∗	1,604.752

Drug/control before induction	Replicates	Drug mean	Control mean	*p* value	% change
Cycloheximide/MQwater	3	5.98197E-07	3.13556E-07	0.317	90.779
Voriconazole/MQwater	3	2.72814E-07	3.41486E-07	0.786	−20.110
Calcimycin/DMSO	3	4.50667E-07	2.51333E-07	0.179	79.310
Sodium Azide/MQwater	3	3.5809E-07	1.69289E-07	0.286	111.526

**Induction**					

Cycloheximide/MQwater	3	1.81389E-06	7.03443E-06	0.327	−74.214
Voriconazole/MQwater	3	3.53236E-06	2.71344E-07	0.391	1,201.803
Calcimycin/DMSO	3	1.58093E-06	9.97417E-07	0.580	58.503
Sodium Azide/MQwater	3	3.41499E-06	2.34075E-07	0.116	1,358.933

PSD values were extracted from three days before and after the induction. Each row represents an individual condition, with PSD values computed on a logarithmic scale (Log10) over a three-day period following induction or control treatment. The analysis was performed exclusively for conditions with sufficient replicates, while controls without fungi were excluded due to the availability of only a single replicate per sample. Additionally, the table reports the percentage change (increase or decrease) in PSD values between the induction and control conditions for each treatment. All data processing and table generation were conducted using RStudio (2024.09.1 Build 394; Document S2). For the fungi/no fungi comparison, f = fungi and nf = no fungi (replicate number). MQwater = Milli-Q water. ∗ = significant *p* value (<0.001).

### Evaluation of the biological origin of electrical signals

**Figure 3:**
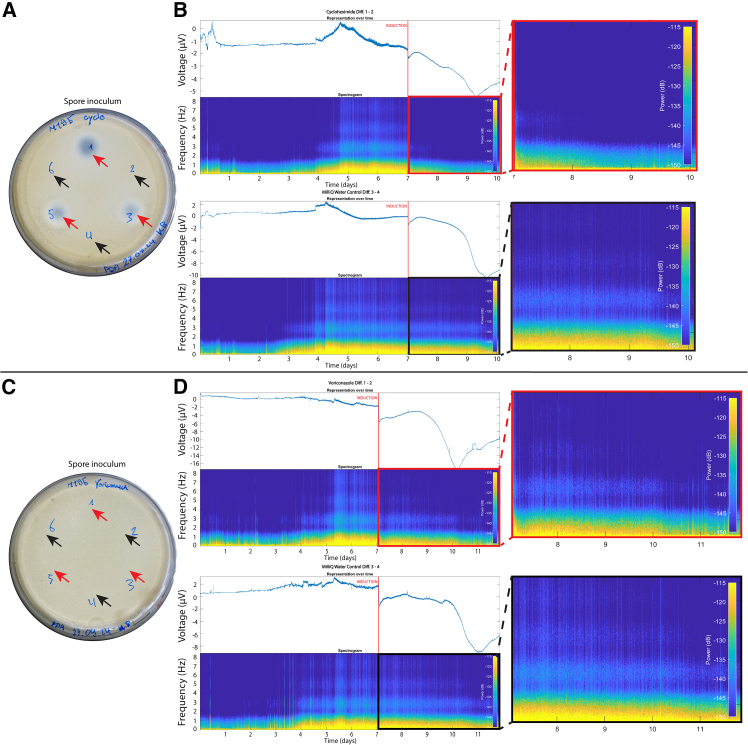
Frequency change analysis of electrical recordings in *Fusarium oxysporum* in response to cycloheximide and voriconazole (A and C) To evaluate the effect of the two biocides, susceptibility tests were performed on spores (for tests on mycelium please see Figures S3 and S4). The site of application of the biocides is shown by the red arrows (cycloheximide in A and voriconazole in C). Black arrows show the addition of Milli-Q water, which is the carrier solvent of the two biocides and acted as a control. (B and D) Examples of voltage plots and their respective interpretation in frequency patterns obtained by STFT analysis (see Figure 2B) for cycloheximide (B) and voriconazole (D). In both cases, the stimuli are shown on the top panel, and the carrier (Milli-Q water) is shown on the bottom panel. The time of application of the stimuli is shown by the red line. The effect of the biocides is shown on the window indicated by the red (biocide) and black (Milli-Q water) squares. The images on the right show a magnification of the frequency pattern after addition of the biocides and Milli-Q water to highlight the effect of the stimuli on the electrical signal produced by the mycelium.

To assess the biological origin of the detected electrical signals, we tested the effect of different chemicals that are known to negatively affect hyphal physiology (Figure 3). For this, we ran experiments with eight electrode pairs at a time. As controls, non-inoculated electrode pairs were also subjected to the applied chemical and chemical carrier (e.g., solvent or Milli-Q water). First, we tested the biocide cycloheximide, confirming that the fungus was sensitive to it by culturing both spores and mycelium in the presence of this antibiotic. Cycloheximide (20 mg/mL) fully inhibited spore germination (Figure 3A) and partially inhibited mycelial growth (Figure S3). The addition of cycloheximide reduced the frequency content under 1.5 Hz to a minimum and eliminated the signals above 1.5 Hz (Figure 3B). In contrast, the addition of the carrier (Milli-Q water) did not alter the frequency content. The frequency content under 1.5 Hz remained unchanged, and the signals above 1.5 Hz (specially the one at 2 Hz) were still observable (Figures 3B and S3). The PSD showed a reduction of 74.21% in the treated pairs compared to the untreated controls but this difference was not significant (Table 1). Next, we tested voriconazole, a second antibiotic. In contrast to cycloheximide, voriconazole (12.5 μg/mL) was shown not to be toxic on a plate assay (Figures 3C and S4). The STFT pattern after addition of voriconazole showed a minor dent in the measurements compared to the carrier (i.e., Milli-Q water; Figures 3D and S4). The PSD increased by 1,201%, but this result was not significant (Table 1). In addition, increased hyphal branching was observed following treatment with voriconazole (Figure S5).

We also tested the antibiotic Calcimycin. In this case, the solvent dimethyl sulfoxide (DMSO) was used as carrier. Since DMSO has been reported to be toxic under some conditions,^44^ we assessed the toxicity of both Calcimycin and DMSO in a plate assay. Calcimycin inhibited spore germination and hyphal growth, whereas DMSO had no obvious impacts on germination or growth (Figure S6). The addition of Calcimycin (1.3 mg/mL) in DMSO also reduced the detected frequencies to a minimum, but DMSO alone produced an identical result (Figure S6). As the effect observed on the signal was not due to DMSO toxicity, it is more likely that DMSO interfered with the gold-plated electrodes.^45^ Three days after the addition of Calcimycin or DMSO, a signal was detectable again in some replicates (Figure S6), suggesting a recovery process. Finally, sodium azide had a substantial impact on spore germination but had a lower impact on established mycelium (Figure S7A). The application of sodium azide (6.5 mg/mL) reduced the signal for about 18 h, before returning to a signal equivalent to the control treatments with Milli-Q water (Figures S7B and S7C). The addition of Milli-Q water in the control resulted in a decrease of the frequency signal in one of the three replicates for a short duration (about 5–6 h) (Figures S7C and S7D), but as all other Milli-Q water controls did not result in a reduced signal, this is likely an artifact. In the case of Calcimycin and sodium azide, the comparison of the PSD showed an increase of the signal after the induction, but the differences were not significant (Table 1).

## Discussion

The recording of voltage changes in fruiting bodies using needle electrodes^31^^,^^32^^,^^33^^,^^34^^,^^35^^,^^36^ triggered the development of techniques to accurately measure voltage in growing mycelium. However, hitherto attempts suffered from flaws in the experimental design, leading to the recording of artifacts and the lack of confidence on the biological origin of the measured signals. These shortcomings have been highlighted by other authors.^37^ To overcome these problems, we established a setup based on the design and construction of the FPC, a printed circuit board in which the differential electrodes were embedded and allowed for the omission of agar-containing media, which can be prone to the development of abiotic electrochemical gradients that can be mistakenly interpreted as true electrical signals.^37^

The use of a Faraday cage was also an essential addition for the improvement of the setup. This feature has been missing in previous studies,^31^^,^^32^ and this has been a common criticism and indicated as an experimental flaw of previous studies.^37^ The addition of the Faraday cage reduced the effect of external noise and artifacts for accurate measurements of voltage changes in the order of microvolts. In previous studies lacking a Faraday cage and employing the same recording instrument, the presence of people walking near the incubator or even the automatic closing of window shades affected voltage measurements.^46^ This issue was eliminated in the current setup.

In addition to an improved experimental setup, we also used a signal analysis methodology that allows us to observe changes in the frequency of electrical signals over time. Previous studies have relied on the characterization of action-potential-like signals directly from the changes in voltage to infer electrical signaling in fungi.^32^^,^^33^ However, the use of a Faraday cage makes it impossible to assess the density of hyphae connected to each electrode, which is a feature required for characterization of action-potential-like signals.^27^^,^^35^ Moreover, action potentials are transmembrane potentials that arise between the intracellular and extracellular space and are generally measured with microelectrodes placed inside living cells. Instead, extracellular electrodes record field potentials (i.e., local voltage changes) when placed close to or in contact with excitable cells. This type of indirect measure of cellular activity reflects the synchronous electric behavior of multiple cells.^37^^,^^47^ To obtain a signal that corresponded to this synchronous behavior, the changes in voltage were broken down into their frequency components using an STFT. This analysis is akin to the analysis of electroencephalographic signals in studies of human physiology,^48^ but in this case, it was applied to the changes in voltage produced upon colonization of the second electrode in the FPC. The STFT, an adaptation of the fast Fourier transform (FFT) algorithm,^49^ is useful in this case because it allows for the extracting of the non-stationary frequency content from noisy signals,^50^ which better correspond to those expected by the non-coordinated expansion of a mycelial network.

A set of signature frequencies above 1.5 Hz were recorded in the time frame in which colonization of the second electrode was to be expected according to previous studies with the same fungus.^38^ The observed range of frequencies (1.5–8 Hz) is consistent with previous studies using internal electrodes, in which frequencies ranging from 0.5 to 5 Hz were recorded.^29^ For comparison, the animal central neuronal system acts at much higher frequencies (up to 200–300 Hz).^51^ However, frequencies in other organs such as the heart (1 Hz for the normal heartbeat) or the intestine (slow waves 0.05–0.2 Hz) are lower.^52^ Similarly, electrical communication in plants depend on signals at lower frequencies.^2^^,^^53^ For instance, the fast cell movement required for the closing of marginal tentacles in the carnivorous plant *Drosera capensis*^54^ is triggered by two action potentials in less than 30 s (4 Hz).^55^ In contrast, other types of signals are thought to use frequencies below 0.2 Hz.^56^ The range of frequencies observed for the mycelium of *F. oxysporum* appears to be compatible with the speed of signal integration required in a digestive system (i.e., intestinal movement), a heartbeat, or to the one existing for fast cell movement in plants. This offers potential for comparative studies across organisms. However, it is still unclear if the signal conveys information or if it is the result of periodic ion redistribution in growing hyphae. Nevertheless, if this mechanism allows intracellular communication, it could contribute to the fast adaptation and the dynamic response of the mycelium to external stimuli, in combination with other processes such as bulk transport of chemical signals.^57^

The comparison of the effect of different biocides with different mechanisms of action on mycelial growth or spore germination suggests that the recorded signals have a biological origin. The effect of cycloheximide, which was confirmed to be toxic in culture assays, suggests that the signal is generated by the active growth of the fungus. Cycloheximide is a biocide of bacterial origin that affects protein synthesis, and it is widely used as a fungicide.^58^ Cycloheximide was toxic, and its addition reduced the frequency content under 1.5 Hz and eliminated the signals above 1.5 Hz. The rapid decrease of electrical signals confirms that those are not merely noise, but rather a physiological indicator reflecting the cellular activity of the mycelium. Voriconazole is also a commonly used fungicide that stops ergosterol production, resulting in an initial stop of growth followed by the destruction of the membrane.^59^ However, voriconazole was not toxic for *F. oxysporum* on a plate assay. In contrast to cycloheximide, voriconazole resulted only on a minor dent in the signal that could be the result of temporary cytosol leaking in the affected hyphae. However, mechanisms ensuring survival, such as the plugging of septal pores,^20^^,^^21^ could be responsible for the fast recovery and are consistent with the observed hyphal branching.

In the future, the use of the FPC could be complemented by monitoring ion fluctuations at a single hyphal level using fluorescent voltage-dependent dyes or tagged ions in combination with fluorescence microscopy. Likewise, the use of knockout mutants or the silencing of genes coding for putative voltage or ion-gated membrane channels is compatible with the method presented here. All the above will increase our knowledge and understanding of the mechanisms involved in the fluctuations of membrane potentials in fungi and help to determine if these fluctuations contribute to cell-to-cell communication conveying information in fungal networks.

### Limitations of the study

Future experiments should focus on the dissection of the molecular mechanisms generating the detected signals by identifying the ions and ion-gated membrane channels involved in this mechanism of electrical signaling. Unfortunately, the first experiments performed in this direction using Calcimycin were inconclusive due to the interaction of DMSO with the electrodes. Calcimycin is an ionophore for divalent cations across biological membranes and leads to Ca^2+^-dependent cell death in Gram-positive bacteria.^60^ Influx and efflux of Ca^2+^ via Ca^2+^-dependent membrane channels have been involved in thigmotropism and galvanotropism in fungi, relaying topologic information during hyphal growth.^61^^,^^62^^,^^63^ In addition, a study in *Aspergillus nidulans* showed a highly localized Ca^2+^ movement that caused a wave of voltage measurements with variable frequency in response to stress.^64^ Accordingly, we expected that the treatment with Calcimycin would further support the role of Ca^2+^ in fungal electrophysiology. However, Calcimycin was highly toxic, and its effect could not be differentiated from its solvent (DMSO). Future experiments need to consider other solvents to evaluate the role of Ca^2+^ using this antibiotic. Electrical signaling in other microbial systems such as bacterial biofilms has been associated with K^+^ transport, in both Gram-positive and Gram-negative bacteria.^3^^,^^65^^,^^66^ K^+^-gated channels have been detected and characterized in fungi^67^ and could be also tested in the future using the FPCs. Additional experiments could consider testing multiple stimuli such as combining ionophores with sodium azide, which is known to stop the production of ATP^68^ and applied alone had a limited effect on the signal.

## Resource availability

### Lead contact

Further information and requests for resources and reagents should be directed to and will be fulfilled by the lead contact, Pilar Junier (pilar.junier@unine.ch).

### Materials availability

The FPC were produced by prototypos SARL (prototypos.ch).

### Data and code availability

All data are available in the main text or the supplemental information. The original unprocessed data are available in Mendeley Data under the https://doi.org/10.17632/srkxbkh6sp.1. The code for analysis is provided in the Method S1.

## Acknowledgments

We would like to thank Diego Gonzalez for his comments on early versions of this manuscript, Lukas Wick for insightful discussion during the development of the idea, and Ilona Palmieri for technical help.

This research was supported by a Science Focus Area Grant from the US Department of Energy (10.13039/100000015DOE), 10.13039/100006206Biological and Environmental Research (BER), Biological System Science Division (BSSD) under the grant number LANLF59T to P.S.C., and the 10.13039/501100001711Swiss National Science Foundation Grant 310030_212435 to M.K.

## Author contributions

Conceptualization, M.B., S.B., and P.J.; methodology, M.B., S.G., A.J.R., J.M.K., M.K., D.O., S.B., L.P., and P.J.; investigation, M.B., S.G., V.F., and L.P.; visualization, M.B., S.G., G.C., L.P., and P.J.; funding acquisition, A.J.R., P.S.G.C., M.K., S.B., and P.J.; project administration, A.J.R., P.S.G.C., and P.J.; supervision, S.B., L.P., and P.J.; writing—original draft, M.B., S.G., M.K., and P.J.; writing—review & editing, M.B., S.G., A.J.R., J.M.K., M.K., S.B., and P.J.

## Declaration of interests

The authors declare no conflict of interest.

## STAR★Methods

### Key resources table

**Table:** 

REAGENT or RESOURCE	SOURCE	IDENTIFIER
**Biological samples**		

*Fusarium oxysporum*	Laboratory of Microbiology	NEU 195

**Chemicals, peptides, and recombinant proteins**		

Cycloheximide (20 mg/ml),	Thermo Fisher Scientific	357420050
Voriconazole (12,5 μg/ml)	Sandoz®	65064
Calcimycin (1.3 mg/ml)	Merck	CAS 52665-69-7
Sodium azide (6.5 mg/ml)	Merck	45/Kit-No 78374

**Deposited data**		

Raw Data recordings	This work	https://doi.org/10.17632/srkxbkh6sp.1

**Software and algorithms**		

Code for STFT analysis	This work	Document S2

**Other**		

PCB card for recordings	Prototypos.ch	This Work
ADC-24 data logger	https://www.picotech.com	-
Shielded armoured cable D-Sub	(Phoenix Contact, mouser.ch	651-2302146

### Experimental model and study participant details

The experimental model corresponded to *Fusarium oxysporum* Neu 195, a fungal strain obtained from the fungal and bacterial collection of the laboratory of microbiology, University of Neuchâtel, Switzerland.

### Method details

#### Inocula preparation

For the regular maintenance, the organism was plated on Malt Extract Agar (12g/l malt extract [“Support Is Our Success”; SIOS® homebrew, Switzerland, Ref: XE201], 15g/l Technical Agar [Biolife, Japan, Ref: 4110254], 1 l deionized water). *F. oxysporum* was grown for 2 weeks on Potato Dextrose Agar (39 g/l PDA stock powder [CARLROTH®, Germany, Ref: AE92.2]) to induce spore production, spores were then collected washed and re-suspended in 50ml MQ water and stored at 4°C for use. Prior to the experiment, 100 μl of the spore suspension were added to 900 μl of liquid Potato Dextrose Broth (4g/l Potato infusion [MERCK Sigma-Aldrich, Germany, Ref: 52424], 20g/l D[+] Glucose monohydrate [CARLROTH®, Germany, Ref: 6887.1], 1 l deionized water) and well mixed. Then 20 μl of the suspension were deposited on the electrodes (1, 3, 5, 9, 11, 13) at a final concentration of around 350 spores for each drop.

#### Measurement set-up

A high-resolution data logger ADC-24 (Pico Technology, UK) was used to measure voltage potential changes in fungal mycelium. Instead of the sub dermal needles used in previous studies we designed the fungal potential card (FPC), a printed circuit board card with 8 couples of embedded ENIG Electro-less Nickel / Immersion Gold electrodes (Figure S1). The FPC was designed and produced by prototypos SARL (prototypos.ch). The card was connected to the ADC-24 via a shielded armoured cable D-Sub (Phoenix Contact, 651-2302146, mouser.ch) for noise-free recordings. The card was placed inside a tailored-made incubator to keep the system at above 80% humidity. For this, a plastic storage container (l: 21.5, w: 12.5, h: 9.5 cm) was used. A dent (height 2.5 cm) was open on one of the short sides of the box to insert one end of the shielded cable. The remaining space was closed with aquarium silicon to avoid water evaporation and contamination. A 1 cm hole was created on the lid and plugged with a hydrophobic filter to permit gas exchange without reduction of humidity. Inside the incubator, a cylindrical piece of plastic was glued to the bottom to act as a holder of the FPC card (1.5 cm height). Vermiculite was deposited on the bottom of the box (covering one 1 cm) and moistened with sterile MilliQ water to keep high humidity. In addition to the plastic holder, to avoid the card from touching the vermiculate, two rubber caps were fixed between the card and the plastic box to block the FPC in place. A second dent was created in between the lid and the box to pass the grounding cable and attach it from the armoured cable to the Faraday cage. The Faraday cage was made from an aluminium box (ALUTEC aluminium box classic 30, l: 40, w: 30, h: 25 cm). We removed the rubber band separating the lid from the box. A hole of 1.5 cm between the lid and the box was cut to pass the cable inside the box. The conductivity of the Faraday cage was tested from the lid (box closed) to the end of the cable attached to the ADC-24 with the aid of a multimeter.

#### Testing of electrode sizes

Different sizes of electrode were tested (1, 2, 3, 4 mm diameter). A single FPC with the four sizes were produced and tested. Each line was of a different size for a total of 2 couples of electrodes per size. In each case, one of the couples was used as an empty control, on which only the medium (PDB) was added. The second electrode pair was inoculated with a spore suspension on PDB.

### Quantification

#### Signal recording

Once the optimal electrode size to be used in the FPC cards was defined (2 mm; Figure S2), several experiments were designed to evaluate the biological origin of the recorded signals. For this, five different chemicals were used, including four antimicrobial agents: cycloheximide (20 mg/ml), voriconazole (12.5 μg/ml), calcimycin (1.3 mg/ml), and sodium azide (6.5 mg/ml). To add the stimuli, the fungus was inoculated on the first electrode and left to colonize the target electrode for seven days. At this point the ADC-24 was paused to avoid noise recording, because to add the stimuli the faraday cage and the incubator needed to be open. With the aid of a micropipette 2 μl of the stimuli (2.5 μl for Voriconazole to attend the good final concentration of 12.5 ug/ml) were added to one of the empty controls (drops without spores) and to the target drop of 3 inoculated electrode pairs. As an additional control, the solvent in which the compounds were dissolved was added to the second empty control and to the target drop of the remaining three inoculated electrode pairs. The solvent was respectively MilliQ water for cycloheximide, voriconazole, and sodium azide; DMSO for calcimycin. After addition of the stimuli, the ADC-24 recording was restarted immediately and the recording continued for another three days to measure the reaction of the fungus. At the end of the experiment pictures of all the boards were taken with a stereoscope (NIKON SMZ18) to confirm the colonisation of the second electrode.

#### Induction testing on fungal drop setup

As the system is currently a black box with no possibilities to observe directly fungal development, we performed the induction experiment in cell culture treated Petri dishes.^38^ For this the same media and spore suspension were used as described above. As *F. oxysporum* grows faster in this system compared to PCB cards, the stimuli were added after five days and not seven as in the electrical measurement. Microscopical pictures were taken in between the drops and inside the target drops using an inverted microscope (EVOS FL, EVOS M5000, Invitrogen) before the addition of the stimuli and two days after addition to observe the outcome.

#### Short-time Fourier transform

Measurements from the ADC-24 Precision Data Logger (Pico Technology, UK) were acquired using the PicoLog 6 Software (Pico Technology, UK), exported in HDF5 format and analysed in MATLAB R2024a. The signal was sampled with a conversion time per channel of 60 ms and thus a sampling frequency of approximately 17 Hz. The potential on the electrodes was analysed both over time and in a time-frequency representation to analyse the frequency spectrum of the signals as they changed over time. After reading the HDF5 files, Not-a-Number (NaN) values were removed from all channels, caused by the loss of some acquisition samples and by the ADC-24 Precision Data Logger pausing during the stimuli addition phase. For time-frequency domain representation, the short-time Fourier transform (STFT) of each signal was calculated. Calculating a STFT means running small time windows along the signal and calculating the fast Fourier transform (FFT) in each time segment. This gives the frequency content of the signal in the time enclosed in the window. The squared amplitude of the STFT is represented in a time-frequency diagram, i.e., the spectrogram. The STFT was calculated using a Blackman – Harris window with a length of 1024 samples and a window overlap percentage of 80% to improve temporal resolution.

#### Statistical analysis

To conduct a quantitative comparative analysis and associate each spectrum of the different channels with a unique value, the average power was calculated by integrating the power spectral density (PSD). The signals were extracted before and after induction, segmented into intervals of 3 days each. To avoid aliasing phenomena, a low-pass filter was applied that limited the spectrum to 4 Hz; since the spectral content is mainly concentrated below 4 Hz, the signals were subsampled to 8 Hz. The PSD was calculated in each time window (after induction), allowing the average power to be determined. The average power values obtained from the three “Fungi + Induction” channels were subsequently averaged with each other. Similarly, the same operation was performed for the “Fungi + Control” channels. To compare the extracted PSD values, all pairwise comparisons were performed using Welch's t-test, which does not assume equal variances between groups, after confirming the normality of the data using the Shapiro–Wilk test (Table 1). We did not include comparisons with the controls without fungi, as only a single replicate was available for each card in this condition. Additionally, we calculated the percentage increase or decrease in PSD values from the Induction condition to the Control for each experimental condition. Furthermore, to quantify the presence/colonization of the fungus, we pooled similar conditions before the induction and compared those with the controls for all electrodes. This was done as described before and a t-test was used to compare the two groups.

## Supplemental information

Document S1. Figures S1–S7 and Method S1
