# Supplementary material for: Detection of electrical signals in fungal mycelia in response to external stimuli
Source: iScience. 2025 Sep 2;28(10):113484. doi: 10.1016/j.isci.2025.113484 (PMC12483595; doi:10.1016/j.isci.2025.113484)
Supplement: Document S1. Figures S1–S7 and Method S1 [file mmc1.pdf]

iScience, Volume *28*

Supplemental information

Detection of electrical signals in fungal mycelia in response to external stimuli

Matteo Buffi, Silvia Giangaspero, Valerio Foiada, Loïc Puthod, Guillaume Cailleau, Aaron J. Robinson, Julia M. Kelliher, Patrick S.G. Chain, Daniel Oberson, Markus Künzler, Saskia Bindschedler, Lorenzo Pirrami, and Pilar Junier

Supplemental items

This PDF file includes:

Figs. S1 to S7

Method S1- code for analysis

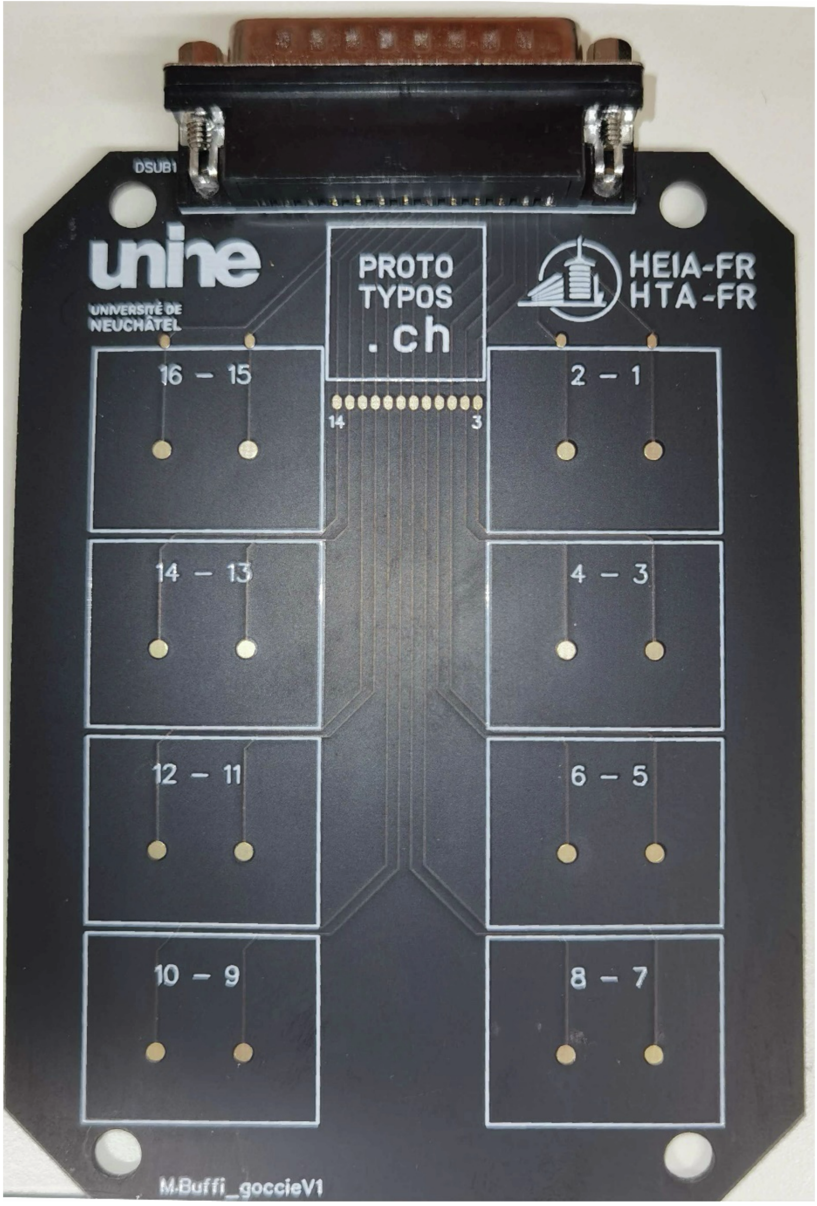

Fig. S1. Image of a printed circuit board (PCB) with embedded gold covered electrodes. Couples of electrodes (1-2, 3-4, 5-6, 7-8, 9-10, 11-12, 13-14, 15-16) are used for the differential measurements resulting in voltage changes between the two electrodes. The PCB were designed by Valerio Foiada, prototypos.ch.

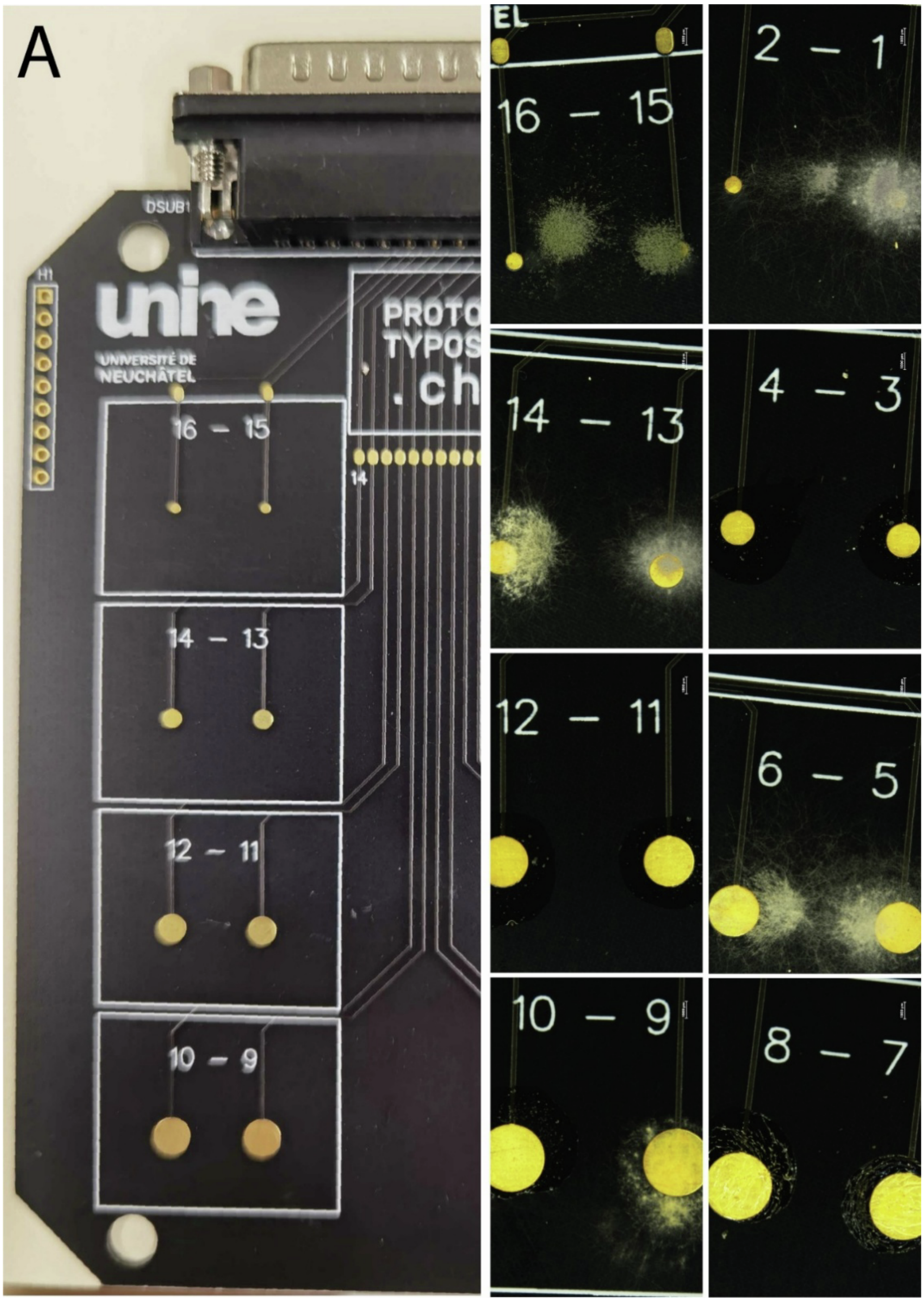

Fig. S2. Testing of optimal electrode size for colonization and recording using circuit board (PCB) with different size electrodes. Empty PCB with embedded gold covered electrodes of different size (from the top electrode sizes of 1, 2, 3 and 4 mm). The pictures on the right show the results obtained during the testing for the growth of *Fusarium oxysporum* on electrodes of different size.

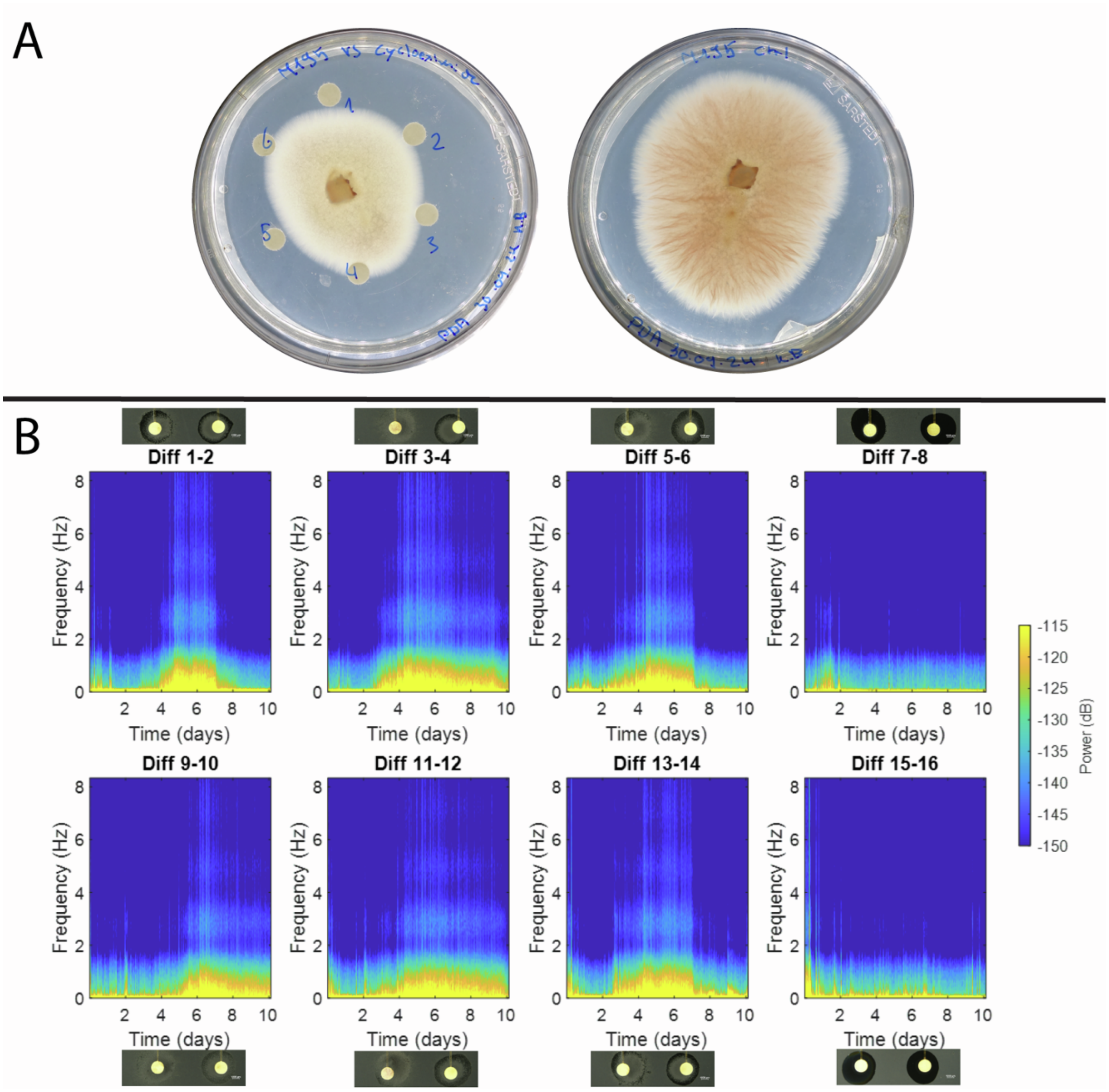

Fig. S3. Effect of cycloheximide on electrical signaling. (**A**) Susceptibility test for cycloheximide and MilliQ water (carrier). The antifungal was applied in the discs 1, 3, and 5 and compared to MilliQ water (discs 2, 4, and 6 (image on the left). Growth on the absence of the discs is shown on the right. (**B**) Differential electrodes colonized by *Fusarium oxysporum* for stimulation with addition of cycloheximide (1-2, 5-6, 13-14, 15-16) or Milli-Q water (3-4, 7-8, 9-10, 11-12), respectively. Electrodes 7-8 and 15-16 were non-inoculated and acted as controls. Pictures were taken with a stereoscope (NIKON SMZ18). All the replicates were performed in the same PCB. The addition of either Cycloheximide (1-2, 5-6, 13-14, 15-16) or Milli-Q water (3-4, 7-8, 9-10, 11-12) was performed at day 7. The images were created with MatLab.

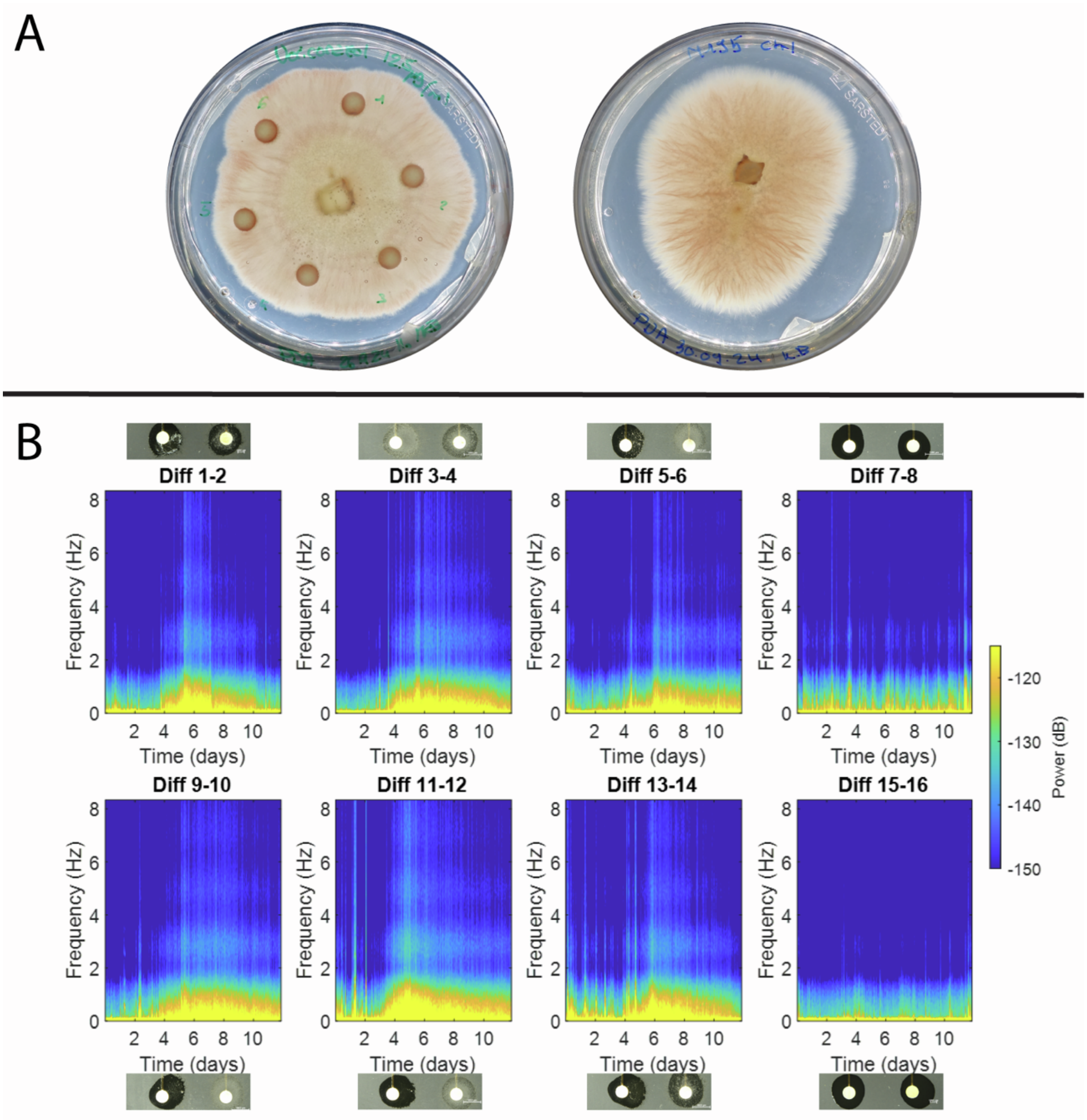

Fig. S4. Effect of voriconazole on electrical signaling. (**A**) Susceptibility test for voriconazole and MilliQ water (carrier). The antifungal was applied in the discs 1, 3, and 5 and compared to MilliQ water (discs 2, 4, and 6 (image on the left). Growth on the absence of the discs is shown on the right. (**B**) Differential electrodes colonized by *Fusarium oxysporum* for stimulation with addition of either voriconazole (1-2, 9-10, 11-12, 15-16) or Milli-Q water (3-4, 5-6, 7-8, 13-14), respectively. Electrodes 7-8 and 15-16 were non-inoculated and act as a control. Pictures were taken with a stereoscope (NIKON SMZ18). All the replicates were performed in the same PCB. The addition of either calcimycine (1-2, 9-10, 11-12, 15-16) or DMSO (3-4, 5-6, 7-8, 13-14) was performed at day 7. The images were created with MatLab.

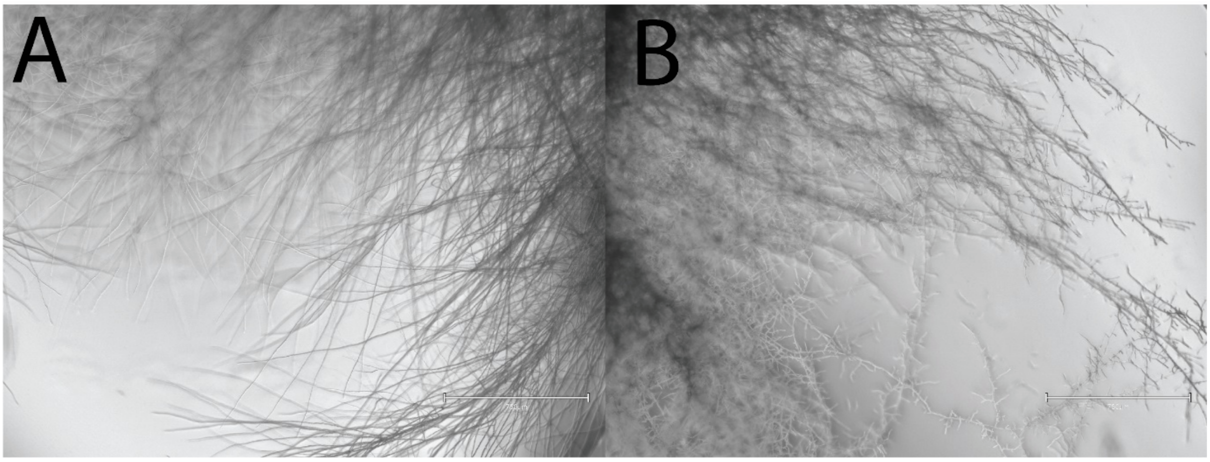

Fig. S5. Effect of voriconazole on growth. Observation of *F. oxysporum* 3 days after the addition of Voriconazole in a drop system. (A) Normal growth after addition of Milli-Q water. (B) Branching of hyphae after addition of Voriconazole. Microscopical pictures were performed with an inverted microscope (Invitrogen, EVOS FL, EVOS M5000).

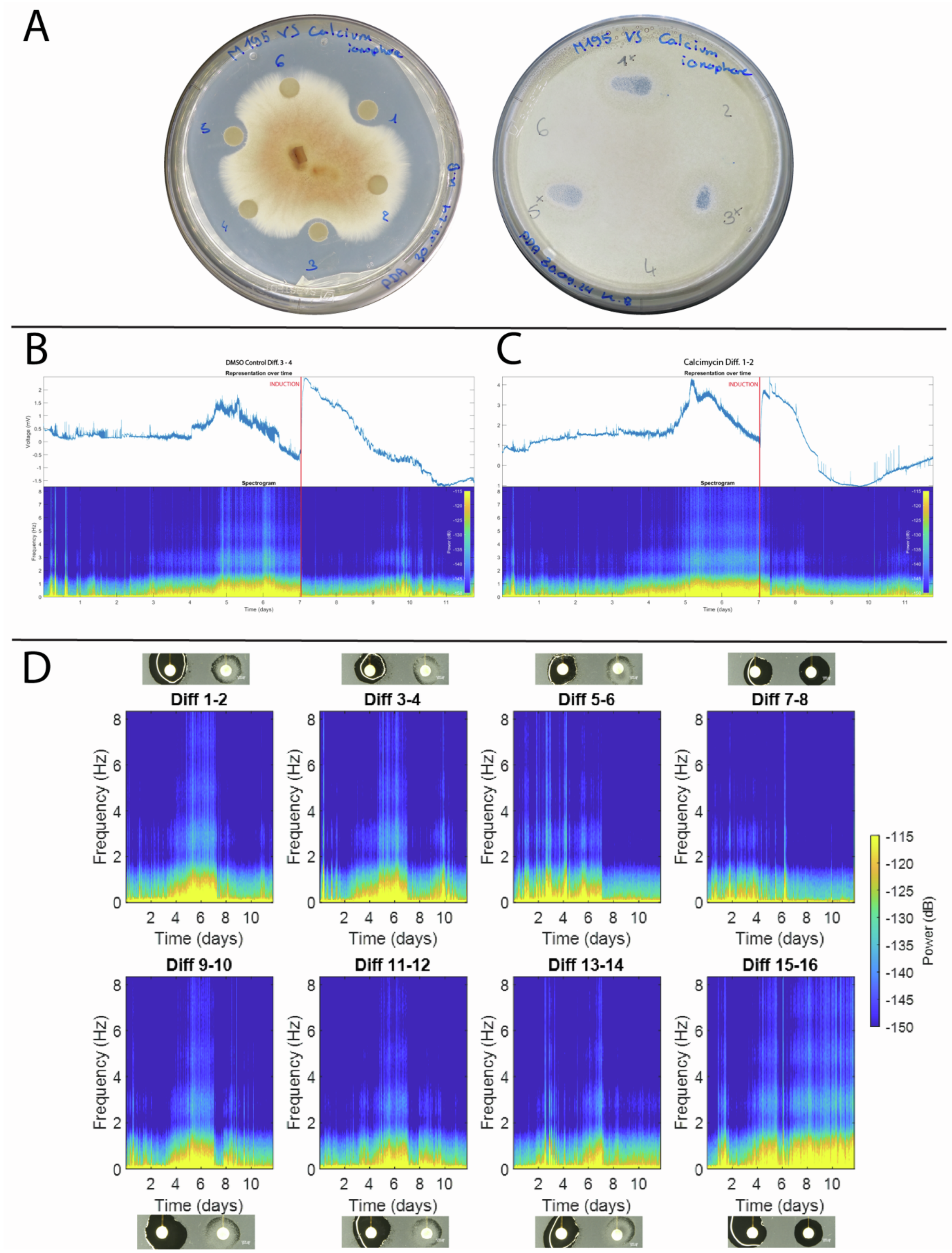

Fig. S6. Effect of calcimycin on electrical signaling. (**A**) Susceptibility test for calcimycin and its solvent (DMSO). On the left *Fusarium oxysporum* was placed using an agar plug on standard potato dextrose agar (PDA); on the right column the fungus was inoculated as spores (final concentration 2 mio) mixed with soft PDA (PDA with only 6 g.l- 1of agar) and poured on a bottom layer of normal PDA. Induction was performed by placing 5 µl of either the tested drug (calcimycin) in 3 different places around the inoculum/center of the Petri dish in positions 1, 3 and 5. The solvent (DMSO) was added in position 2, 4 and 6. The inhibitory effect of calcimycin on both mycelial growth and spore inoculum was observed after 2 days post inoculation. (**B - C**) Frequency change analysis of electrical recordings in *F. oxysporum* in response to calcimycin and DMSO.

Representative recordings for the control carrier (DMSO, **B**) or the corresponding stimulant (calcimycin, **C**) are shown. After 7 days, the recording was stopped (red line) and the stimulant was added to the system. The recording continued after stimulation. (**D**) Differential electrodes colonized by *F. oxysporum* for the testing of either calcimycin (1-2, 5-6, 7-8, 13-14) or DMSO (3-4, 9-10, 11-12, 15-16), respectively. Electrodes 7-8 and 15-16 are non-inoculated and act as a control. Pictures were taken with a stereoscope (NIKON SMZ18). All the replicates were performed in the same PCB. The addition of either voriconazole (1-2, 5-6, 7-8, 13-14) or Milli-Q water (3-4, 9-10, 11-12, 15-16) was performed at day 7. The images were created with MatLab.

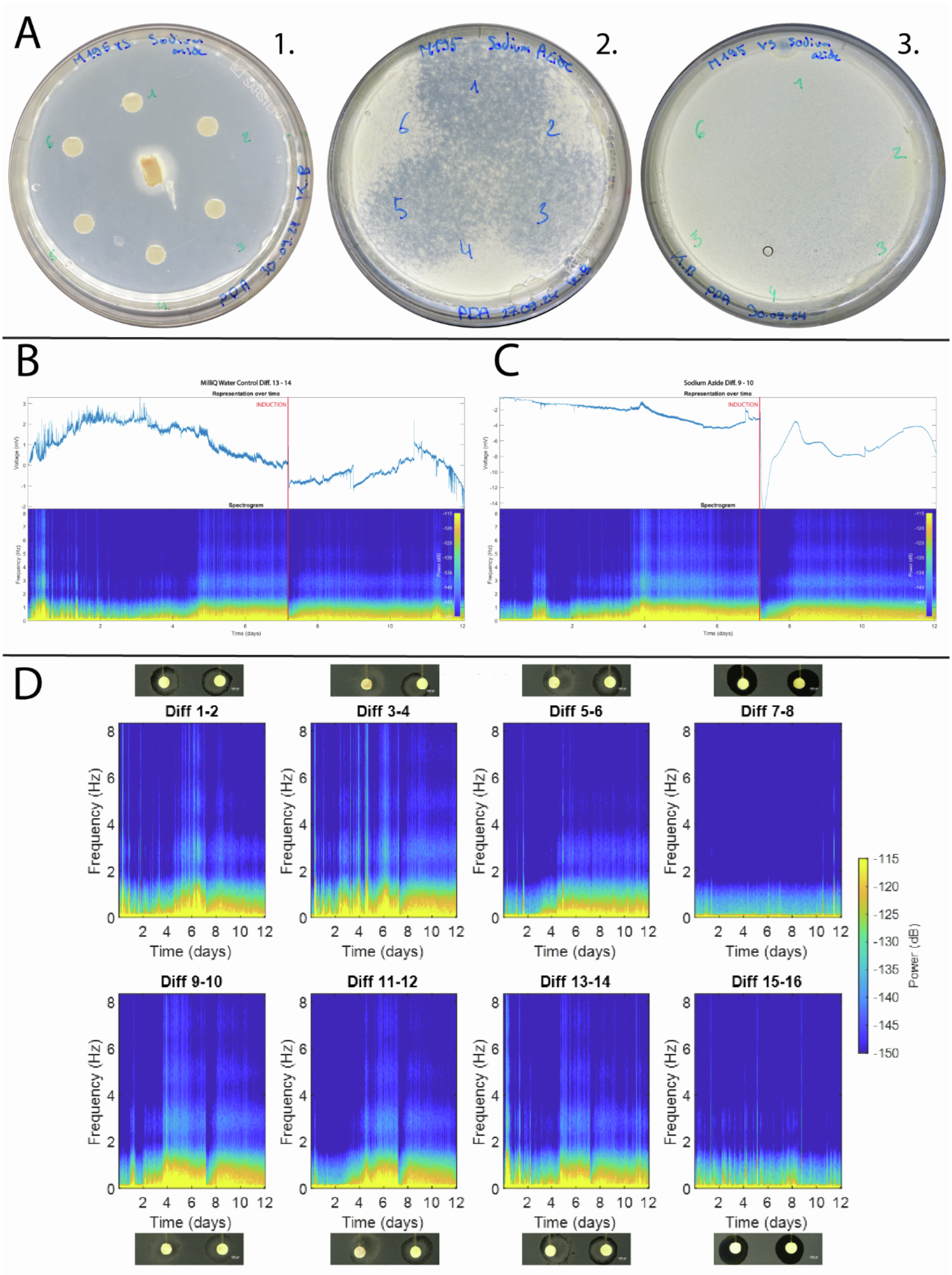

Fig. S7. Effect of sodium azide on electrical signaling. (**A**) Susceptibility test for sodium azide and its solvent (MilliQ water). On the left column (A.1.) *Fusarium oxysporum* was placed using an agar plug on standard potato dextrose agar (PDA); on the right (A.2., A.3.) the fungus was inoculated as spores (final concentration 2 mio) mixed with soft PDA (PDA with only 6 g.l- 1of agar) and poured on a bottom layer of normal PDA. Induction was performed by placing 5 µl of either the tested drug (sodium azide) in 3 different places around the inoculum/center of the Petri dish in positions 1, 3 and 5. The solvent (MilliQ water) was added in position 2, 4 and 6. The stimulant was added with the inoculum for the first two (A.1., A.2.) and at 6 dpi for the last one (A.3.) The inhibitory effect of sodium azide on both mycelial growth and spore inoculum was observed after 2 days post inoculation (A.1., A.2.) or post stimuli addition (A.3.). For controls refer to Supplementary Figure 4A. (**B - C**) Frequency change analysis of electrical recordings in *F. oxysporum* in

response to sodium azide and MilliQ water, representative recordings for the control carrier (MilliQ water, **B**) or the corresponding stimulant (sodium azide, **C**) are shown. After 7 days, the recording was stopped (red line) and the stimulant was added to the system. The recording continued after stimulation. (**D**) Differential electrodes colonized by *F. oxysporum* for the testing with addition of either sodium azide (3-4, 9-10, 11-12, 15-16) or Milli-Q water (1-2, 5-6, 7-8, 13-14), respectively. Electrodes 7-8 and 15-16 are non-inoculated and act as a control. Pictures were taken with a stereoscope (NIKON SMZ18). All the replicates were performed in the same PCB. The addition of either sodium azide (3-4, 9-10, 11-12, 15-16) or Milli-Q water (1-2, 5-6, 7-8, 13-14) was performed at day 7. The images were created with MatLab.

Method S1- Detailed procedure for STFT analysis

Table of Contents

.... 1
Removing NaNs 2
Plot raw data 2
Short - Time Fourier Transform 3


```
clc;
clear;
close all;

infostrings = ["Diff 1-2" "Diff 3-4" "Diff 5-6"...
                "Diff 7-8" "Diff 9-10" "Diff 11-12"...
                 "Diff 13-14" "Diff 15-16"];

dt        = 60e-3;                  % temporal resolution (s)
fs        = 1/dt;                   % sampling frequency (Hz)

% -------------------------------------------------------------------------
% CHANGE
%
% 1) FILENAME with PATH TO THE HDF5 FILE
%
% 2) SUBSTANCE with NAME OF INJECTED SUBSTANCE
% -------------------------------------------------------------------------

substance = "Cycloheximide";

filename = "";

% -------------------------------------------------------------------------

%h5disp(filename)

data.diff1_2 = h5read(filename,'/Differenziale 1 - 2');
data.diff3_4 = h5read(filename,'/Differenziale 3 - 4');
data.diff5_6 = h5read(filename,'/Differenziale 5 - 6');
data.diff7_8 = h5read(filename,'/Differenziale 7 - 8');
data.diff9_10 = h5read(filename,'/Differenziale 9 - 10');
data.diff11_12 = h5read(filename,'/Differenziale 11 - 12');
data.diff13_14 = h5read(filename,'/Differenziale 13 - 14');
data.diff15_16 = h5read(filename,'/Differenziale 15 - 16');

fields = fieldnames(data);
numchann = length(fields);
N = length(data.(fields{1}));
time = linspace(1, N*dt-dt, N); % in seconds
```

Removing NaNs

```
% Find the channel with the most NaN values
maxNaN = -1;
maxNaNChannel = '';
for i = 1:numchann
    numNaN = sum(isnan(data.(fields{i})));
    if numNaN > maxNaN
        maxNaN = numNaN;
        maxNaNChannel = fields{i};
    end
end

disp(['Channel with the most NaN values: ', maxNaNChannel]);

nanIndices = find(isnan(data.(maxNaNChannel)));

% Remove NaN indices from other channels to match lengths
for i = 1:numchann
        data.(fields{i})(nanIndices) = [];
end

time(nanIndices) = [];
N = length(time);

disp('NaN values have been removed and channels have been aligned.');

% %% Downsampling at 8 Hz
%
% fsNew = 8;          % new sampling frequency (Hz)
% dtNew = 1/fsNew;    % new temporal resolution (s)
%
% factorDown = round(fs/fsNew);
% timeDown = downsample(time, factorDown);
% Ndown = length(timeDown);
%
% % downsampling
% for i = 1 : numchann
%     dataDown.(fields{i}) = downsample(data.(fields{i}), factorDown);
% end
```

Plot raw data

```
figure
for i = 1 : numchann
    plot(time/(60*60*24), data.(fields{i})*1e3);
    hold on;
end
title(substance)
legend(infostrings)
xlabel('Time (days)'); ylabel('Voltage potential (mV)');
axis tight
```

```
grid on;
ax = gca;
ax.GridAlpha = 0.3;  % Set grid transparency to make it lighter
ax.XGrid = 'on';
ax.XMinorGrid = 'on';
ax.XMinorTick = 'on';
ax.XAxis.MinorTickValues = time(1)/(60*60*24):1/4:time(end)/(60*60*24); % Set
minor ticks for every 6 hours
```

Short - Time Fourier Transform

```
% Parameters
win = blackmanharris(1024);          % Increased window length
Overlap = 800;                       % Increased overlap
FFTLength = 2048;                    % Increased FFT length

for i = 1:numchann

    figure
    tile_l = tiledlayout(2, 1, 'TileSpacing', 'none', 'Padding', 'compact');

    % Plot the time-domain signal (downsampled)
    ax1 = nexttile;
    plot(time/(60*60*24), data.(fields{i})*1e3);
    axis tight;
    ylabel('Voltage (mV)');
    title('Representation over time');
    set(gca, 'XTick', []);

    % Calculate and plot the spectrogram
    ax2 = nexttile;
    [~,F,T,P] = spectrogram(data.(fields{i})-mean(data.(fields{i})), win,
Overlap, FFTLength, fs, 'yaxis', 'power');

    % Smooth the spectrogram with a 2D Gaussian filter
    sigma = 2; % Standard deviation of the Gaussian kernel
    P_smoothed = imgaussfilt(10*log10(P + eps), sigma);

    imagesc(T/(60*60*24), F, P_smoothed);

    axis xy;
    ylabel('Frequency (Hz)');
    xlabel('Time (days)');
    title('Spectrogram');
    % Modified Colorbar Placement
    h = colorbar('east', 'Color', 'white');
    h.Label.String = 'Power (dB)';
    clim([-150 -115]);

    % Add overall title
    title(tile_l, sprintf('%s - %s', substance, infostrings(i)), 'FontSize',
12);
```

```
end

% Create a single figure for all STFT plots
figure

for i = 1:numchann
    % Calculate the spectrogram for each channel
    subplot(2, 4, i);
    [~, F, T, P] = spectrogram(data.(fields{i}) - mean(data.(fields{i})),
win, Overlap, FFTLength, fs, 'yaxis', 'power');

    % Smooth the spectrogram with a 2D Gaussian filter
    sigma = 4; % Standard deviation of the Gaussian kernel
    P_smoothed = imgaussfilt(10*log10(P + eps), sigma);

    imagesc(T/(60*60*24), F, P_smoothed);
    axis xy;
    ylabel('Frequency (Hz)');
    xlabel('Time (days)');
    title(infostrings(i));

    % Emphasize color differences and smooth representation
    clim([-150 -115]); % Set the range of colors for better emphasis
end

% Adjust colorbar to cover all subplots
colorbar;

% Add colorbar label
h = colorbar;
h.Label.String = 'Power (dB)';

% Add an overall title to the figure
sgt = sgtitle(substance);
sgt.FontSize = 10;
```

Published with MATLAB® R2024b
